# Cardiac Niche Influences the Direct Reprogramming of Canine Fibroblasts into Cardiomyocyte-Like Cells

**DOI:** 10.1155/2016/4969430

**Published:** 2015-11-23

**Authors:** Giacomo Palazzolo, Mattia Quattrocelli, Jaan Toelen, Roberto Dominici, Luigi Anastasia, Guido Tettamenti, Inès Barthelemy, Stephane Blot, Rik Gijsbers, Marco Cassano, Maurilio Sampaolesi

**Affiliations:** ^1^Translational Cardiomyology Laboratory, Stem Cell Biology and Embryology Unit, Department of Development and Regeneration, KU Leuven, Herestraat 49 O&N4 Bus 814, 3000 Leuven, Belgium; ^2^Laboratory of Stem Cells for Tissue Engineering, IRCCS, San Donato Hospital, San Donato, Milan, Italy; ^3^Organ Systems Development and Regeneration Unit, Department of Development and Regeneration, KU Leuven, Herestraat 49 O&N4 Bus 814, 3000 Leuven, Belgium; ^4^Laboratory of Clinical Chemistry, Public Health Corporation of Legnano, Magenta Hospital, 20013 Magenta, Italy; ^5^Department of Biomedical Sciences for Health, University of Milan, Segrate, Milan, Italy; ^6^Laboratoire de Neurobiologie, Ecole Nationale Vétérinaire d'Alfort, 94704 Maisons-Alfort, France; ^7^Laboratory of Molecular Virology and Gene Therapy, Department of Pharmaceutical and Pharmacological Sciences and Leuven Viral Vector Core, KU Leuven, Leuven, Belgium; ^8^School of Life Sciences, EPFL, 1015 Lausanne, Switzerland; ^9^Human Anatomy Unit, Department of Public Health, Experimental and Forensic Medicine, University of Pavia, Via Forlanini 8, 27100 Pavia, Italy

## Abstract

The Duchenne and Becker muscular dystrophies are caused by mutation of dystrophin gene and primarily affect skeletal and cardiac muscles. Cardiac involvement in dystrophic GRMD dogs has been demonstrated by electrocardiographic studies with the onset of a progressive cardiomyopathy similar to the cardiac disease in DMD patients. In this respect, GRMD is a useful model to explore cardiac and skeletal muscle pathogenesis and for developing new therapeutic protocols. Here we describe a protocol to convert GRMD canine fibroblasts isolated from heart and skin into induced cardiac-like myocytes (ciCLMs). We used a mix of transcription factors (GATA4, HAND2, TBX5, and MEF2C), known to be able to differentiate mouse and human somatic cells into ciCLMs. Exogenous gene expression was obtained using four lentiviral vectors carrying transcription factor genes and different resistance genes. Our data demonstrate a direct switch from fibroblast into ciCLMs with no activation of early cardiac genes. ciCLMs were unable to contract spontaneously, suggesting, differently from mouse and human cells, an incomplete differentiation process. However, when transplanted in neonatal hearts of SCID/Beige mice, ciCLMs participate in cardiac myogenesis.

## 1. Introduction

Ischemic heart disease is one of the leading causes of death worldwide and so far therapeutic approaches are limited [1].

Because of the negligible regenerative ability, the heart has been considered for nearly a century as a terminal differentiated postmitotic organ [2]. Although this concept is currently outdated, the heart is not able to heal itself after injury by any native processes, and fibrotic scars replace necrotic tissue. This stiffens the heart tissue and prevents the normal contractility of cardiomyocytes. Cardiac fibroblasts (CFs) are widely involved in the heart healing process and represent one of the largest cell populations in the myocardium [3]. For this reason, CFs have been identified as ideal cell source for* in vivo* direct conversion approaches [4].

The discovery of MyoD, as master gene for skeletal muscle differentiation [5], generated a broad interest in cell reprogramming by using defined factors. Unfortunately, for cardiac differentiation, a single master gene such as MyoD is not known yet. Ieda et al. reported that the forced expression of three exogenous transcription factors (Gata4, Mef2c, and Tbx5) in neonatal cardiac and dermal fibroblasts is sufficient for the conversion to cardiomyocyte-like cells* in vitro* [6]. After this first elegant study, several groups reported similar results using different transcription factors and microRNA [7–10]. Recently, two groups reported the conversion of fibrotic scar tissue into induced cardiomyocytes-like cells* in vivo* through retroviral delivery of GATA4, MEF2C, and TBX5 (GMT) transcription factors [11, 12]. In the same year, Olson's group demonstrated an improvement of the cardiac differentiation rate combining the GMT with Hand2 both* in vitro* and* in vivo* [10]. Similar results have been achieved lately on human fibroblasts using the same protocol [13, 14].

These discoveries hold a great promise for the treatment of heart chronic diseases where the invading fibrotic tissue could be replaced by contractile cardiomyocytes. Muscular dystrophies (MDs) are a group of inherited diseases caused by mutations in the Dystrophin Glycoprotein Complex. Patients affected by MDs, in particular Duchenne and Becker muscular dystrophy, who survived to the third decade of life are affected by cardiomyopathy and heart failure is the main cause of death for these patients [15–17]. The heart degeneration and remodelling lead to the formation of subepicardial fibrosis of the inferolateral wall [18] that could represent the ideal target for direct lineage reprogramming to cardiomyocyte lineage. Among the widely used animal models of DMD, the golden retriever muscular dystrophy (GRMD) dog is considered the closest model to the human disease in terms of size and pathological onset of the disease. In fact, the clinical course of GRMD dogs is characterized by progressive muscle wasting, degeneration, fibrosis, and shortened lifespan [19–21]. Cardiac involvement in GRMD dogs has been demonstrated by electrocardiographic studies, revealing a progressive cardiomyopathy similar to DMD patients [22–24]. In this respect, GRMD dog is a useful model for the development of new therapeutic protocols to improve cardiac function [25].

In this study we aim to evaluate the direct lineage conversion strategy (GATA4, MEF2C, TBX5, and HAND2) on cardiac and skin fibroblasts isolated from a large animal model of Duchenne muscular dystrophy (GRMD). Canine-induced cardiac-like myocytes (ciCLMs) expressed late cardiac markers genes, immature sarcomeric structures, and engrafting ability* in vivo*.

## 2. Material and Methods

### 2.1. Isolation and Culture of Skin Canine Fibroblasts

Canine skin samples from GRMD dogs were obtained from fresh abdominal skin biopsies and isolated as previously shown [26]. Obtained canine fibroblasts were kept in culture in DMEM supplemented with 15% FBS and antibiotics. The medium was replaced every two days. Cells were frozen, after being cultured for one week, and then plated for lentiviral transduction.

### 2.2. Isolation and Culture of Cardiac Canine Fibroblasts

Left ventricular heart biopsy from a deceased GRMD dog was chopped into small pieces and plated on a tissue culture dish in DMEM : 199 (4 : 1), 15% FBS and antibiotics. Cardiac fibroblasts spread out from the minced heart after few days and one week later cells were frozen. The medium was replaced every two days.

### 2.3. Cell Culture

Bj-1 and canine cardiac and skin fibroblast were maintained in DMEM supplemented with 5 mM L-glutamine, 5 mM sodium pyruvate, 100 IU/mL penicillin, 100 *μ*g/mL streptomycin (all from Invitrogen), and 20% fetal bovine serum (Gibco). Neonatal rat cardiomyocytes were used for the preparation of the induction medium (IM) and for coculture with transduced fibroblasts. Neonatal rat cardiomyocytes were isolated using the Neonatal Cardiomyocyte isolation kit from Worthington Biochemical Corporation (Lakewood, NJ, USA) and cultured in DMEM 10% FBS (Invitrogen, Carlsbad, CA, USA). Medium was replaced and frozen every day and subsequently used in the preparation of the induction medium (IM). Primary cell cultures were maintained in a humidified incubator at 37°C with 5% CO_2_.

### 2.4. Plasmid Cloning and Expansion

Lentiviral vectors were prepared by using standard procedures. Bacterial stocks were grown in Luria-Broth medium ON at 37 degrees. All vectors preparations were obtained using a GenElute HP Endotoxin-Free Plasmid Maxiprep Kit (Sigma) and Plasmid DNA was quantified using Nanodrop system.

The plasmid carrying the human transcription factors (GATA4, MEF2C, TBX5, and HAND2) and *α*-MyHC-GFP cardiac differentiation reporter gene were subcloned into the lentiviral vector pCHMWS carrying different resistance genes (Figure 2(a)).

### 2.5. Lentivirus Production

HEK 293 cells were seeded at density of 6 × 10^6^ in a 10 cm tissue culture dish and at the day after being transfected, using Lipofectamine 2000 (Invitrogen), with nine micrograms of the individual transfer vectors. Cells were kept in OptiMem; after six hours the medium was replaced to 10 mL of fresh DMEM with 10% FBS and antibiotics. The medium containing lentiviral particles was harvested after 36 hours of transfection, filtered with 0,45 *μ*m cellulose filter.

### 2.6. Viral Transduction and Differentiation

Skin, cardiac, and Bj-1 fibroblasts were plated at density of 1 × 10^5^/cm^2^ on Nunc culture dishes (ThermoFisher Scientific) and transduced with fresh lentiviral supernatant (MOI = 50) mixed with polybrene at final concentration of 4 *μ*g/*μ*L (Sigma). The day after transduction with the mix of transcription factors, medium was replaced with induction medium (IM), containing 10% conditioned medium (obtained from neonatal rat cardiomyocyte culture), DMEM/199 (4 : 1), 10% FBS, 5% horse serum (HS), antibiotics, nonessential amino acids, essential amino acids, B-27, insulin-sodium selenite-transferrin, vitamin mixture, and sodium pyruvate (Invitrogen) [10]. Eukaryotic selection antibiotics (Blasticidin 5 *μ*g/mL, Hygromycin 100 *μ*g/mL, and Zeocin 200 *μ*g/mL) were added to the IM the second day after transduction and kept in culture for one week. Induction medium was replaced every two days.

### 2.7. Real Time PCR

Total RNA was extracted from cells using RNeasy Mini Kit (Qiagen) and 1 *μ*g was reverse transcribed to cDNA by Superscript III Reverse Transcriptase (Life Technologies). Real Time PCR was performed using Platinum SYBR Green qPCR SuperMix-UDG and Mastercycler ep realplex (Eppendorf). qPCR experiments were performed using the following primers:

 hGATA4: Fw5′-tctcagaaggcagagagtgtg; Rv5′-ggttgatgccgttcatcttgt;
 hMEF2C: Fw5′-aagaagggccttaatggctgt; Rv5′-atctcgaagttgggaggtgga;
 hTBX5: Fw5′-gcatggagggaatcaaagtg; Rv5′-cttcgttttgggattaaggcc;
 hHAND2: Fw5′-atgagtctggtaggtggttttcc; Rv5′-catactcggggctgtaggaca;
 dGATA4: Fw5′-cgggctatcatcccactatg; Rv5′-gcgactggctgacagaagat;
 dMEF2c: Fw5′-cccgatgcagacgattca; Rv5′-caaaattgggaggtggaaca;
 dTBX5: Fw5′-ccttctaccgctcaggctac; Rv5′-caggctgggcacaggctca;
 dHAND2: Fw5′-ccgctaaccgcaaggaga; Rv5′-cgtcttgatcttggagagtttg;
 ACTC1: Fw5′-tcccatcgagcatggtatcat; Rv5′-ggtacggccagaagcataca;
 CNX43: Fw5′-ccagttgagattccactcagt; Rv5′-gttgagtaccacctccactga;
 TNNT2: Fw5′-atgtctgacgtagaagaggag; Rv5′-atcaccttccgcgttggtctc;
 MYH6: Fw5′-gccctttgacattcgcactg; Rv5′-ggtttcagcaatgaccttgc.

### 2.8. Western Blotting

Cells were collected 14 days after transduction and resuspended in Ripa buffer (Sigma) supplemented with 10 mm NaF (Sigma), 0.5 mm NaOrthovanadate (Sigma), 1 : 100 Protease Inhibitor Cocktail (Sigma), and 1 mm PMSF (Sigma). Western blot analysis was performed on 40 *μ*g of cell lysate, using anticardiac-TnnI (Tnni3; Abcam; ab19615) 1 : 100 as primary antibody and secondary HRP-conjugated antibodies (Santa Cruz), 1 : 500. Bands were detected and pictured at Bio-Rad GelDoc by means of Pico substrate (Thermo Fisher Scientific; Dura substrate for DYS analysis); densitometry analyses were carried on gels loaded, blotted, and detected in parallel by means of QuantityOne software (Bio-Rad).

### 2.9. Immunocytochemistry and Flow Cytometry Analyses

Cells were fixed 14 days after transduction in 2% PFA, permeabilized with 0.2% Triton, and blocked with donkey serum (Sigma). Cells were then stained overnight at 4°C with different primary antibodies. Antibody dilutions were the following: anti-Vimentin (Sigma; V6630) 1 : 200, anti-Ki67 (sigma; ab15580) 1 : 200; anti-GFP (Abcam; ab545) 1 : 500; anti-sarcomeric *α*-actinin (*α*SA; Abcam; ab9465) 1 : 200; anti-connexin 43 (Santa Cruz; sc-9059) 1 : 300; MyHC (MF20; Hybridoma) 1 : 3; cardiac troponin T (Abcam; ab10214) 1 : 200.

Secondary fluorescents antibodies (Alexa-Fluor; 1 : 500) were used for detection. Nuclei were stained with Hoechst 33342 (Sigma).

Flow cytometry analyses were performed according to standard procedures with the following antibodies: anti-CD90/Thy1 antibody (FITC) (Abcam; ab124527); anti-cardiac troponin T (Abcam; ab10214).

Cardiac troponin T expression was analyzed by intracellular staining; cells were fixed with 2% PFA, blocked with donkey serum (Sigma), and permeabilized by fixation/permeabilization (BD Cytofix/Cytoperm).

### 2.10. *In Vivo* Xenotransplant Experiment

GHMT GFP positive cells were sorted 7 days after transduction using AriaIII FACS and 5 × 10^4^ were injected directly in the heart of 2-day-old SCID/Beige-mice pups. After 2 weeks, mice were sacrificed and the whole heart was embedded in OCT (Sakura, Tissue-Tek) and cut as 10 *μ*m sections using a cryostat (Leica). Sections were fixed in 4% PFA, permeabilized by solution PBS-BSA1%-Triton X-100, and immunostained by antibodies against dystrophin (NCL-DYS2-NCL-DYS3; Novocastra; 1 : 50), myosin heavy chain (MF20; Hybridoma; 1 : 3), and GFP (Abcam; ab5450).

### 2.11. Statistical Analysis

Values are expressed as mean ± SD, and when two groups were compared, unpaired Student's *t*-test was used. When more than two groups were compared, a two-way analysis of variance (ANOVA) was used and a probability of less than 5% (*p* < 0.05) was considered to be statistically significant (Bonferroni post hoc test). Statistical significance of the differences between the percentage values was assessed by the Kruskal-Wallis one-way ANOVA on ranks, with a Bonferroni-Dunn post hoc test, and significance was scored when *p* < 0.05 for both tests.

## 3. Results

### 3.1. Characterization of Fibroblast Populations

**Figure 1:**
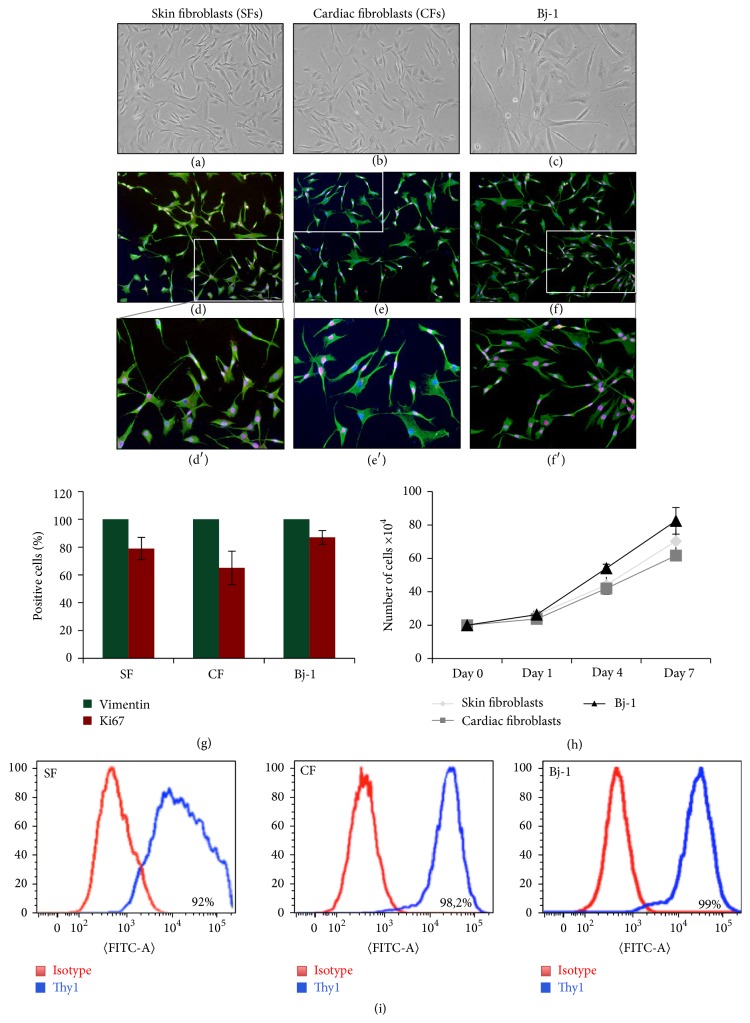
Canine primary culture characterization. Skin (a) and cardiac (b) canine fibroblasts showed similar morphology to human Bj-1 cells (c) and express in similar fashion Vimentin (in green) and Ki67 (in red) (d–f). The percentage of Vimentin and Ki67 positive cells was similar in the three cell populations (g). Growth curves of canine fibroblasts (*n* = 5/fibroblast type) showed comparable proliferation ability with respect to Bj-1 fibroblasts (h). (i) Examples of FACS analysis revealing that canine fibroblast primary cultures highly expressed Thy1 (CD90), similarly to Bj-1 fibroblasts.

In the last years, several groups reported the possibility to induce direct reprogramming from adult somatic cells to other lineages through the overexpression of a set of transcription factors or miRNA [6, 10, 27–31]. In this study we tested whether the same four cardiac transcription factors, GHMT, shown previously to direct the reprogramming on neonatal human foreskin fibroblasts [14], were able to reprogram fibroblasts isolated from GRMD dogs. According to those previous studies, canine fibroblasts were considered for the reprogramming protocol due to their easy access and ability to proliferate* in vitro*. We characterized canine fibroblasts for their morphology (Figures 1(a)–1(c)), expression of Vimentin as mesodermal marker, and Ki67 as proliferative marker (Figures 1(d)–1(f)) and cell growths have been analysed (Figure 1(g)). In addition, Bj-1 human fibroblasts, a commercially available adult human dermal fibroblast cell line, were used as control. The three fibroblast populations resulted 100% positive for Vimentin and fibroblasts derived from skin and cardiac biopsies were, respectively, 79% and 65% positive for Ki67, slightly lower than Bj-1 (87%). In order to avoid possible contamination of progenitor cells, known to be more susceptible to reprogramming, we employed cell-sorting strategy in our freshly isolated primary cultures to isolate homogenous fibroblast populations. FACS analysis showed that cells were homogenously positive for CD90 (Thy1) (Figure 1(i)), similarly to Bj-1 cell line. CD90 positive cells were subjected to the reprogramming protocol.

### 3.2. Transcription Factors Overexpression

**Figure 2:**
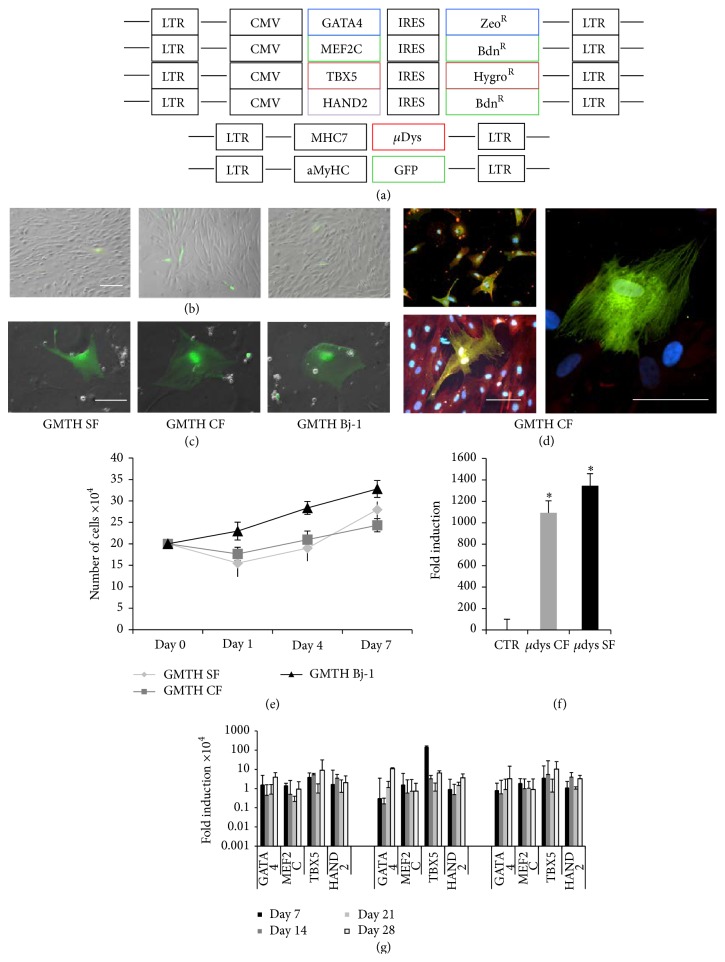
Viral transduction and exogenous gene expressions. (a) Schematic representation of the lentiviral vectors carrying the human sequence of the transcription factor genes (GATA4-MEF2C-TBX5-HAND2), the microdystrophin gene, and the GFP reporter gene. (b) The GFP reporter gene under the control of the *α*-MyHC promoter was already expressed 48 hours after transduction. (c) Fibroblasts underwent morphology changes 7 days after transduction and become flattened and binucleated cells expressing GFP. (d) Immunofluorescence analysis on GHMT CF alone (top left panel) and in coculture with neonatal rat cardiomyocytes (bottom left panel), with antibodies against GFP (green) and myosin heavy chain (red), shows double positive cells and single positive cells for GFP (right panel). (e) Cell growth curve after transduction shows a decrease of cell proliferation (*n* = 5/cell type). (f) Fold induction of microdystrophin (*μ*dys) mRNA expression in transduced cells compared to the control; *n* = 5  ^*∗*^ *p* < 0.01, CF and SF versus controls (CF and SF transduced with empty vector). (g) Exogenous transcription factors expression (*n* = 5) after transduction shows high induction levels during the 28 days of differentiation.

To determine the best set of transcription factors able to convert fibroblast to cardiac-like cells, we generated lentiviruses carrying different genes involved in cardiac differentiation including GATA4, HAND2, MEF2C, TBX5, MESP1, and BAF60c. A cardiac muscle specific promoter (alpha myosin heavy chain, *α*MyHC) was used to drive expression of GFP in our cell system, as gene tracer for cardiac differentiation. We infected Bj-1 cells with different mix of lentiviral vectors and we evaluated the conversion potential by GFP expression. In our hand, the best set of genes resulted in being GHMT (data not shown). Indeed, the overexpression of GHMT has been shown to be sufficient for the reprogramming of tail tip and cardiac fibroblast [10] and human fibroblasts [14] to cardiac-like cells. In the following experiments, coding sequence genes for GATA4, HAND2, MEF2C, and TBX5 (GHMT) were cloned into lentiviral vectors carrying different gene resistance (Figure 2(a)) and used to infect CD90+ fibroblasts from skin (SF) and cardiac (CF) GRMD biopsies and Bj-1 human cell line. Cells were infected with a MOI of 50 and after 2 days few GFP positive cells were detected in all samples (Figure 2(b)). Then the medium was replaced with the differentiation medium and cells were maintained in culture up to 28 days. GFP positive cells underwent drastically morphological changes, resulting in flattened and binucleated cells similar to foetal cardiomyocytes (Figure 2(c)). GFP positive GHMT CFs were sorted and counterstained, alone (Figure 2(d), upper left panel) or in coculture with rat cardiomyocytes (Figure 2(d), lower left panel), for myosin heavy chain in order to confirm the specificity of our differentiation protocol. GFP and myosin heavy chain (MyHC) colocalized in the majority of the cells (Figure 2(d), left upper panel). However, we could also find occasionally GFP positive cells negative for MyHC (Figure 2(d), right panel), suggesting possible leakiness of the *α*MyHC promoter. At day 1 from the infection, a small population of skin and cardiac canine fibroblasts died; however, they maintained a comparable proliferation rate to GHMT Bj-1 fibroblasts in the following days (Figure 2(e)). With the prospective of a possible cell therapy and functional tests after cell conversion, we transduced GHMT CFs and GHMT SFs with lentiviral vectors carrying microdystrophin (*μ*dys) gene, and *μ*dys expression was evaluated via qRT-PCR in transduced cells (Figure 2(f)). As shown in Figure 2(g), infected cells maintained a high and comparable expression of GHMT transcription factors over the 28 days of differentiation.

### 3.3. Evaluation of the Conversion of Fibroblast to ciCLMs

**Figure 3:**
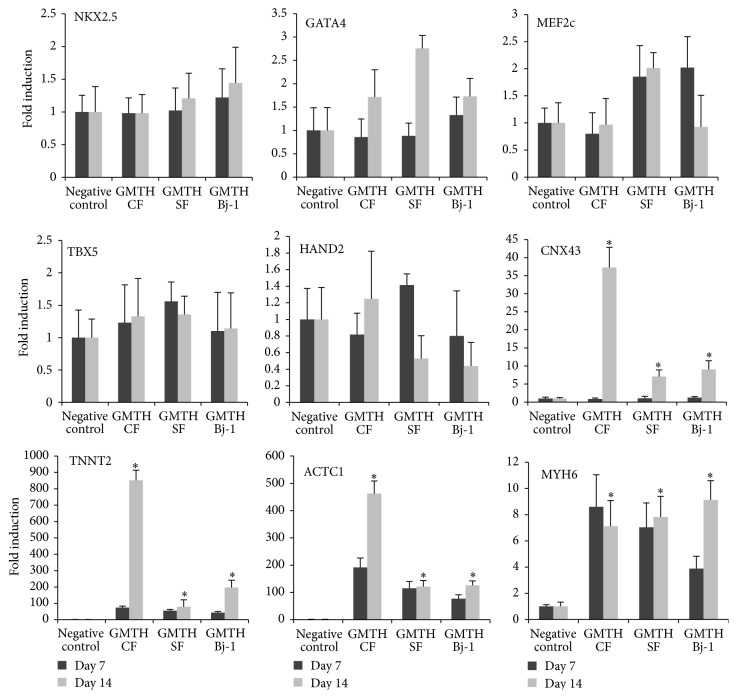
Characterization of GHMT transduced cells. qRT-PCR was performed at day 7 and at day 14 from transduction. Early endogenous cardiac marker genes (Nkx2.5, Gata4, and Mef2c-Tbx5-hand2) and late cardiac marker genes (CNX43, TNNT2- ACTC1 and MYH6) were analysed. Fold induction of mRNA expression in transduced cells compared to the controls (cells transduced only with *α*-MHC-GFP vector). The expression of late cardiac markers identified canine-induced cardiac-like myocytes (ciCLMs) in the transduced cell populations; *n* = 5, 2-way ANOVA test, ^*∗*^ *p* < 0.01.

**Figure 4:**
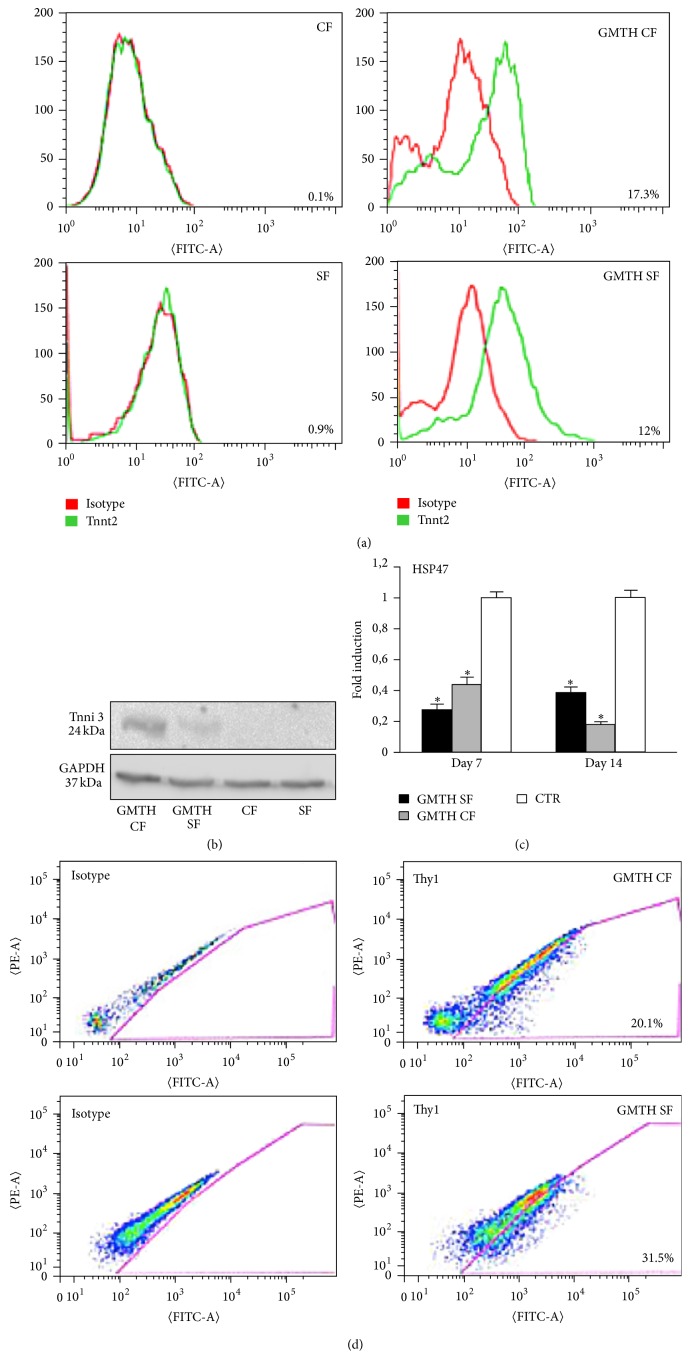
Expression of cardiac and fibroblast markers in ciCLMs. (a) Examples of intracellular FACS analysis for the isolation of Tnnt2+ cells (green) and isotype controls (red) in ciCLMs after 14 days of differentiation. (b) Example of Western blot analysis after 14 days of differentiation showed the presence of cardiac troponin I as a late cardiac marker protein in ciCLMs derived from both GHMT CF and GHMT SF cells. Densitometry quantification performed with ImageJ software showed that Tnni3 was 3,2-fold more expressed in GHMT CF compared to GTMH SF. Troponin I was absent in CF and SF transduced with *α*-MHC-GFP vector. (c) Expression of the fibroblast marker HSP47, in transduced cells compared to control cells (transduced with only *α*-MHC-GFP vector). HSP47 expression strongly decreases in ciCLMs after 7 and 14 days of differentiation compared to the controls; *n* = 5, 2-way ANOVA test, ^*∗*^ *p* < 0.01. (d) Examples of FACS analysis on ciCLMs confirmed a reduced number of Thy1 (CD90) + cells after 7 days of differentiation.

**Figure 5:**
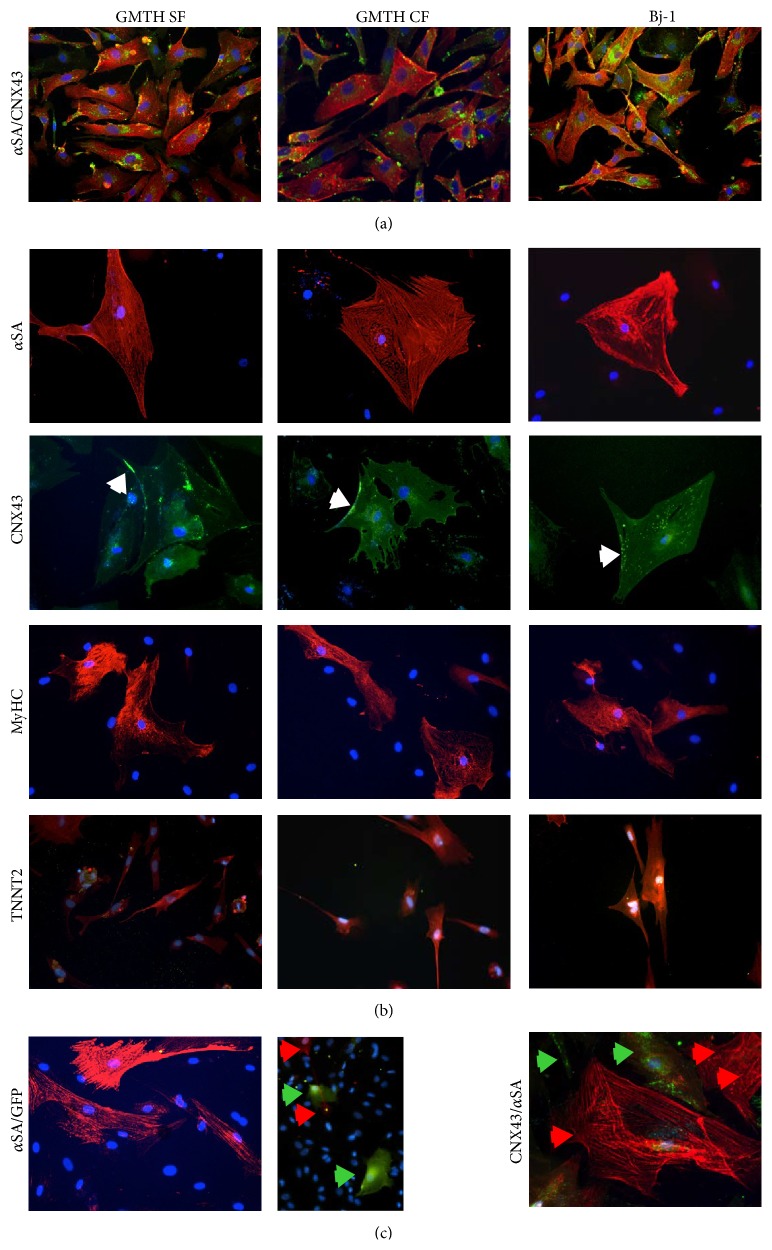
Localization of cardiac markers in ciCLMs. (a) Alpha sarcomeric actinin (red) and connexin 43 (green) were present in ciCLMs in similar amount to reprogrammed Bj-cells. (b) Immunofluorescence analysis revealed the expression of alpha sarcomeric actinin (*α*SA, red), connexin 43 (CNX43, green; white arrows indicate CNX43 expression on the cell membrane), myosin heavy chain (MyHC, red), and cardiac troponin T (red) in ciCLMs after 14 days under differentiating conditions. (c) ciCLM cells did not express cardiac markers homogenously. A few cells expressing *α*SA were GFP negative and vice versa (left panels) and occasionally CNX43 (green arrows) and *α*SA (red arrows) were mutually exclusively expressed (right panel).

To evaluate the conversion of fibroblasts into cardiac myocyte-like cells, we analysed the expression of early (MEF2c, NKX2.5, GATA4, TBX5, and HAND2) and late (CNX43, TNNT2, ACTC1, and MYH6) cardiac markers at different time points (Figure 3). After 14 days in differentiation medium, we detected an increase of the expression of late cardiac markers. On the contrary, we did not detect a consistent fold change in the expression of endogenous early cardiac markers. It is likely that due to the constitutive expression of the exogenous transcription factors genes involved in the first steps of cardiomyogenesis were repressed. Interestingly we found that CNX43, TNT and ACTC1 were significantly more highly expressed in GHMT CFs compared to GHMT SFs and Bj-1, suggesting that fibroblasts derived from a cardiac niche could retain a higher conversion potential to cardiac myocytes-like cells. Moreover, confirmatory results were obtained with FACS (Figure 4(a)) and Western blot analysis (Figure 4(b)). When cells were stained for the cardiac marker Tnnt2 and evaluated via FACS analysis (Figure 4(a)), we detected 17.3% and 12% Tnnt2+ cells, respectively, in GHMT CFs and GHMT SFs. In addition, Western blot analysis with anti-TnnI3 antibodies revealed a higher amount of troponin I protein in GHMT CFs compared to GHMT SFs. On the contrary, troponin I was not detected in the negative control (Figure 4(b)). Consistent with the upregulation of cardiac specific genes during the conversion, we found an important decrease in the expression level of the fibroblast markers HSP47 (Figure 4(c)) and CD90 (Figure 4(d)) in GHMT CFs and GHMT SFs compared to controls. Although between 20 and 30% of cells still expressed Thy1 (CD90), we cannot exclude that the reprogramming protocol altered their fibroblast phenotype. Up to 95% of converted cardiomyocytes survive and we managed to keep them in culture for 30 days. Immunofluorescence analysis was then performed (at day 14) to further elucidate the protein organization in the ciCLMs. GHMT CF, GHMT SF, and Bj-1 cells stained positive for alpha sarcomeric actinin (*α*SA), connexin 43 (CNX43), myosin heavy chain (MyHC), and cardiac troponin T (TNNT2) (Figures 5(a) and 5(b)). The results obtained from immunofluorescence analysis seem to represent a spectrum of cardiac reprogramming, where ciCLMs induced from adult cardiac and skin fibroblasts are composed of different cell types, previously referred to as types A, B, and C [10]. However, we could not observe more organized sarcomeres in ciCLMs at day 30 compared to day 14 and indeed no beating cells were detected.

### 3.4. Xenotransplantation of ciCLMs in SCID/Beige Mice

**Figure 6:**
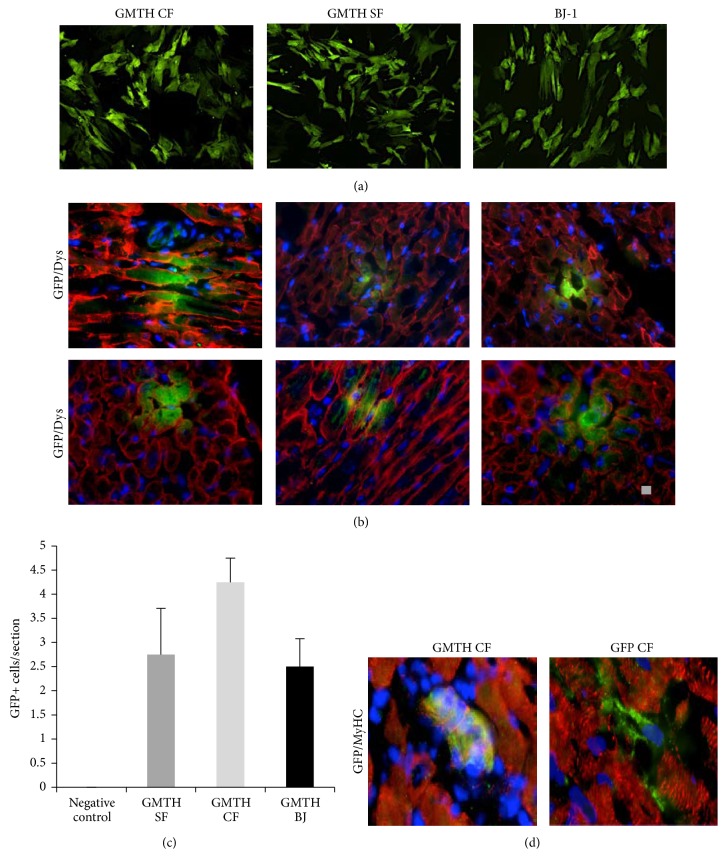
ciCLMs* in vivo* injection. (a) Immunofluorescence analysis for GFP expression ciCLMs after cell sorting for GFP and before injections. (b) 2 × 10^5^ GFP positive cells were injected directly in the heart of 2-day-old pups. After 14 days pups were sacrificed and heart sections were prepared and double stained with antibodies against dystrophin (red) and GFP (green). (c) Average number of ciCLMs GFP positive cells per section; *n* = 3. (d) Double staining with antibodies against myosin heavy chain (red) and GFP (green).

In order to evaluate the ability to contribute to the heart development, we set up a xenotransplant model of the GHMT cells into developing heart of 1-day-old SCID/Beige pups, as previously reported [32]. GFP positive cells were sorted after 7 days from GHMT transduction (Figure 6(a)) and 50.000 cells of each cell type were injected directly in the heart of 12 pups. Three weeks after the injection, mice were sacrificed and hearts were explanted and immunostained for GFP and dystrophin. Immunofluorescence analysis revealed that ciCLMs were able to engraft in the growing heart as shown in Figure 6(b). Interestingly, we found that ciCLMs generated from CF have a higher engraftment capacity (Figures 6(c) and 6(d), left panel) compared to those generated from SF. On the contrary, injected GFP positive CF cells, used as negative control, were confined in the interstitial area (Figure 6(d), right panel).

## 4. Discussion

Duchenne and Becker muscular dystrophies are a group of inherited diseases that affect both skeletal and cardiac muscles. In particular, adolescence patients tend to develop dilated cardiomyopathy that could result in arrhythmia, fatigue, shortness of breath, and swelling of the legs and feet, and that could lead to heart failure and sudden death [33]. Cardiac fibroblasts (CFs) are involved in the healing and remodeling process of the heart resulting in scar tissue formation with limited contractile properties. A possible therapeutic approach would be to convert CFs into functional cardiac myocytes. Indeed several studies showed the possibility to generate cardiomyocytes by direct reprograming with specific transcription factors [10–14]. In this context, we isolated cardiac and skin fibroblasts from GRMD dogs, a large animal model for Duchenne muscular dystrophy, and attempted to reprogram them into canine-induced cardiac-like myocytes (ciCLMs) using a conversion protocol recently described [6, 14]. Our results showed that the overexpression of four cardiac transcription factors (GHMT) is sufficient to convert cardiac and skin canine fibroblasts into ciCLMs. In order to generate a reliable protocol, we employed cell-sorting technology to obtain CD90+ cells as a starting cell population from fresh isolated cells. This cell population was up to 100% positive for Vimentin and HSP47. As already described in literature [34], the healthiness and freshness of the starting population represent a critical step for obtaining good rate and quality of conversion to ciCLMs. After isolation, cells were highly proliferative as demonstrated by cell growth curves and the Ki67 staining. However, after some passages in culture, some signs of senescence were observed. To preserve proliferative potential, cells were expanded for 10 days after isolation and then frozen for later reprogramming experiments. In our study, we included Bj-1 fibroblast cells as controls and they showed comparable conversion efficiency with canine fibroblasts. Nevertheless, the use of human transcription factors could represent a further limitation in our reprogramming method. GFP+ cells were already detectable 48 hours after transduction with GHMT and *α*MHC-GFP and at 14 days cells become flattened and binucleated, expressing late cardiac markers such as CNX43, TNNT2, ACTC1 and MYH6. Early endogenous cardiac genes did not show evident modulation during the reprograming protocol, highlighting the fact that cardiac differentiation occurred differently from cardiac embryonic development. In all our attempts, no beating cells were detected from cells subjected to the direct conversion protocol as previously reported for human fibroblasts [35]. We speculate that this could be due to two different issues in our direct reprogramming protocol. First, we used human transcription factor cDNA, although the human transcription factor sequences used for transduction are well conserved among mammalian species and the similarity between canine and human sequences is between 96% and 98%. Second it is well recognized that isolated cells from newborn pups retain a higher plasticity and regeneration potential compared with cell derived from adult animals. However, in our experiments we used fibroblasts isolated from skin and heart biopsies of adult deceased GRMD dogs, since we could not obtain cardiac fibroblasts from pups due to ethical restrictions for large animal models. We speculate that those limitations could have affected the efficiency of our reprogramming protocol. Another important issue emerging in the recent literature is the stoichiometric ratio of reprogramming factors.

Indeed, using a mixture of viruses expressing individual factors has heterogeneous and unmanageable ratio of reprogramming factor expression among infected fibroblasts, which results in variable and low reprogramming effectiveness.

To further investigate the conversion potential efficiency of canine fibroblasts subjected to reprogramming, we analysed the expression of late cardiac markers by qPCR, the percentage of Tnnt2+ cells via FACS, and quantified Tnni3 protein via Western blot. Interestingly our results showed for the first time that GRMD canine cardiac fibroblasts have higher conversion ability to ciCLM cells compared to skin and dermal human fibroblasts. Indeed, qPCR analysis showed that GHMT CFs expressed higher level of CNX43, TNNT2 and ACTC1 after 14 days of cardiac differentiation. In addition, 17.3% of GHMT CFs and 12% of GHMT SFs were positive for Tnnt2. Densitometry quantification showed that Tnni3 was 3,2-fold more expressed in GHMT CF compared to GTMH SF. Furthermore, both GHMT CF and GTMH SF downregulated the fibroblast markers, HSP47 and CD90, after 14 days of conversion. Consistent with literature, IF analysis revealed that ciCLM cells do not express all the cardiac markers homogenously, since they could represent a spectrum of cardiac differentiation [10].

Finally, sorted GFP+ ciCLM cells at day 7 of conversion, injected into the heart of SCID/Beige newborn pups, were able to engraft the developing heart and expressed dystrophin and MyhC. On the contrary, injected GFP-CFs remain confined in the interstitial area. Although the mechanisms and stoichiometric effects in cell fate specification are still largely unknown, this latter result highlights that GHMT forced expression is sufficient to generate cardiac progenitors from somatic adult canine cells able to integrate into the cardiac niche.

## 5. Conclusions

In this study we provided evidences that canine fibroblasts are converted to immature cardiac myocytes (ciCLMs) with the GHMT reprogramming protocol. We showed that ciCLMs express cardiac late markers and ability to engraft in a developing heart in xenotransplantation experiment. Moreover, we found that fibroblasts isolated from cardiac tissue are more prone to cardiac conversion than skin fibroblasts, suggesting that an epigenetic regulation shapes cardiac cell fate. Polycistronic constructs to express homogenously each factor as recently reported for human fibroblasts [36] could improve this methodology to obtain a large number of canine ciCLMs.

## Nonstandard Abbreviations and Acronyms

*α*SA: *α*-sarcomeric actinin
Actc1: *α*-cardiac actin
Bdn^r^: Blasticidin resistance gene
ciCLMs: Canine-induced cardiac-like myocytes
CF: Cardiac fibroblasts
DMD: Duchenne muscular dystrophy
DMEM: Dulbecco's modified eagle medium
Ki67: Antigen identified by monoclonal antibody Ki67
FACS: Fluorescent activated cell sorting
FBS: Fetal bovine serum
GATA4: GATA binding protein 4
GMT: Mix of 3 transcription factors, GATA4, MEF2C, and TBX5
GHMT: Mix of 4 transcription factors, GATA4, HAND2, MEF2C, and TBX5
GFP: Green fluorescent protein
GRMD: Golden retriever muscular dystrophy
HS: Horse serum
Hygro^r^: Hygromycin resistance gene
IF: Immunofluorescence
IM: Induction medium
MDs: Muscular dystrophies
MEF2C: Myocyte enhancer factor type 2C
MYH6: Cardiac myosin heavy chain *α*
MyoD: Myogenic differentiation
Nkx2.5: NK2 transcription factor related, locus 5
qPCR: Quantitative polymerase chain reaction
SCID: Severe combined immunodeficient disease
SFs: Skin fibroblasts
TBX5: T-box transcription factor
TnnI3: Cardiac troponin I
WB: Western blot
Zeo^r^: Zeocin resistance gene.
